# Artificial Intelligence and Deep Learning in Sensors and Applications

**DOI:** 10.3390/s24103258

**Published:** 2024-05-20

**Authors:** Shyan-Ming Yuan, Zeng-Wei Hong, Wai-Khuen Cheng

**Affiliations:** 1 Department of Computer Science, National Yang Ming Chiao Tung University, Hsinchu 300093, Taiwan; 2 Department of Information Engineering and Computer Science, Feng Chia University, Taichung 40724, Taiwan; zwhong@fcu.edu.tw; 3 Faculty of Information and Communication Technology, Department of Computer Science, Universiti Tunku Abdul Rahman, Kampar 31900, Malaysia; chengwk@utar.edu.my

## 1. Introduction

To effectively solve the increasingly complex problems experienced by human beings, the latest development trend is to apply a large number of different types of sensors to collect data in order to establish effective solutions based on deep learning and artificial intelligence [1,2,3,4]. This not only creates a huge demand for sensors, providing business opportunities, but also creates new challenges for the development of sensor devices and their related applications [5,6]. These technological developments that combine AI and sensors are being actively used in various application fields such as healthcare, manufacturing, agriculture and fisheries, transportation, construction, environmental monitoring, etc.

For instance, in environmental monitoring, sensors integrated with deep learning and AI algorithms possess the capability to swiftly analyze extensive datasets, identifying patterns, anomalies, and trends in real-time [7,8]. Consider weather forecasting, where sensors driven by AI can gather data from various sources like satellites, weather stations, and drones, enabling more precise predictions of weather patterns. Through deep learning models, sensors can dynamically adjust and incorporate new data, thereby refining their predictive accuracy over time. Additionally, in industrial settings, sensors enhanced with AI play a crucial role in optimizing manufacturing operations by monitoring equipment health, forecasting potential failures, and scheduling maintenance preemptively [9,10,11,12]. This approach reduces operational downtime and enhances overall efficiency.

In this context, the Special Issue “Artificial Intelligence and Deep Learning in Sensors and Applications” collected high-quality original contributions on new developments in AI (specifically deep learning) and sensor technology in various fields, as well as sharing ideas, designs, data-driven applications, and production and deployment experiences and challenges. The call for papers for this Special Issue included topics such as applications and sensors for manufacturing, machinery, and semiconductors; smart applications and sensors for architecture, construction, buildings, e-learning; recommendation systems; applications and sensors for autonomous vehicles, traffic monitoring, and transportation; object recognition, image classification, object detection, speech processing, human behavior analysis; and other related sensing applications [13,14].

## 2. Overview of Published Papers

All submissions were evaluated based on their technical excellence, resulting in the selection of ten research articles, and two reviews on weed detection using deep learning and medical XAI, for inclusion in this Special Issue. Below, all of the contributions are listed, followed by a short summary of each contribution.

In contribution 1, the author contributes to the field by exploring the integration of AI and deep learning for enhancing lung cancer screening through sensors, such as CT scans, enriched with clinical data. By comparing single-modality (imaging data alone) and multimodality approaches (combining imaging with clinical data), the study underlines the importance of leveraging multiple data types for improved diagnostic accuracy. Specifically, it showcases the application of ResNet18 for 3D CT imaging analysis and random forest algorithms for clinical data evaluation, demonstrating a significant leap in performance with multimodal data fusion. This work aligns with the journal’s focus by illustrating how AI and deep learning can optimize sensor data utilization in healthcare applications, pushing the boundaries of early cancer detection capabilities.

In contribution 2, the innovative C2RL framework introduces a convolutional-contrastive learning approach that integrates with reinforcement learning to enhance the generalization capabilities of agents in varied environments, especially those captured through high-dimensional image sensors. This paper’s approach addresses the challenge of leveraging strongly augmented sensor data without compromising the reinforcement learning process. By demonstrating its effectiveness in the DeepMind Control suite, the paper contributes to the journal’s theme by showcasing how AI and deep learning techniques can enhance the interpretability and utility of sensor data in complex, dynamic systems, paving the way for more robust AI agents that can operate in diverse and unpredictable real-world scenarios.

In contribution 3, TransNet leverages transformer architecture to introduce a novel approach for point cloud sampling, addressing the computational complexities associated with direct usage of dense point cloud data from sensors. This contribution is particularly relevant to the journal’s interests, as it illustrates the application of AI and deep learning in optimizing sensor data processing. By implementing self-attention mechanisms, TransNet not only reduces computational demands, but also enhances precision in tasks requiring detailed spatial analysis, such as autonomous driving and robotic navigation, highlighting the potential of advanced AI models in improving the efficiency and accuracy of sensor-based applications.

In contribution 4, DCFF-MTAD presents a dual-channel feature fusion model for anomaly detection in multivariate time-series data, a common output from various sensors. This paper’s approach, combining spatial STFT and graph attention networks, exemplifies the integration of AI to enhance sensor data analytics for predictive maintenance and monitoring of complex systems. It aligns with the journal’s focus by demonstrating the potential of deep learning for extracting and fusing features from sensor-generated data to identify anomalies, offering valuable insights for applications in industrial IoT, environmental monitoring, and more, where accurate and timely anomaly detection is crucial.

In contribution 5, MTGEA addresses the challenge of aligning sparse point cloud data from radar sensors with skeleton data from Kinect sensors for human activity recognition, a critical task in smart home systems. The paper’s development of a multimodal two-stream GNN framework exemplifies innovative use of deep learning to enhance sensor data compatibility and application efficiency. This work is highly relevant to the journal, as it showcases the fusion of data from diverse sensors through AI to improve accuracy in privacy-preserving human activity recognition, contributing to the advancement of smart home technologies and healthcare monitoring systems.

In contribution 6, the author explores semi-supervised learning techniques for semantic segmentation, leveraging perturbed unlabeled inputs to enforce consistency in dense prediction tasks. By focusing on one-way consistency and introducing a novel perturbation model, this research advances the application of AI in processing and interpreting sensor data, especially in real-time and low-power scenarios. The findings are pertinent to the journal’s scope, as they highlight how deep learning can reduce reliance on extensively labelled datasets in dense prediction, facilitating more efficient and scalable implementations of AI in semantic segmentation tasks using data from visual sensors for applications in autonomous driving, surveillance, and robotics.

In contribution 7, the author contributes significantly to the realm of AI and sensor applications by addressing the vulnerabilities of deep-learning-based face recognition systems to adversarial patch attacks. By using Generative Adversarial Networks (GANs), the study proposes a method that successfully implements black-box attacks, enhancing the realism and applicability of security assessments in systems that rely on facial recognition sensors. This contribution is vital for developing robust face recognition technologies that are increasingly integrated into various security and personal identification applications, ensuring they can resist real-world adversarial threats.

In contribution 8, the introduction of a real-time, on-chip audio super-resolution system tailored for bone-conduction microphones marks a notable advancement in sensor technology and AI applications. This paper’s development of lightweight deep-learning models for enhancing audio quality directly addresses the limitations of existing sensor technology in noisy environments. By optimizing these models for low-power, real-time processing on embedded systems, the study provides a practical solution that can be integrated into next-generation hearing aids and communication devices, leveraging AI to significantly improve the user’s experience.

In contribution 9, the research enhances the application of AI in industrial sensors by introducing a deep learning framework capable of detecting anomalies in the network traffic of industrial control systems. The use of multi-attention residual blocks to refine the feature extraction process represents a forward leap in improving the accuracy and reliability of anomaly detection in critical infrastructure. This paper’s methodology contributes to safer, more secure industrial operations by leveraging deep learning to interpret complex sensor data streams effectively.

In contribution 10, the author contributes to the field of AI and sensor applications through their innovative use of convolutional LSTM networks combined with a multi-head attention mechanism, specifically designed to enhance traffic flow predictions. By effectively analyzing and predicting traffic conditions using vast arrays of sensor data, this approach helps with optimizing traffic management and urban planning. The integration of spatial and temporal data analyses in a unified deep learning model exemplifies how AI can harness sensor data to facilitate smarter, more efficient city infrastructure.

In contribution 11, this systematic literature review aggregates and synthesizes the use of AI in agricultural sensors for weed detection, presenting an overview of the state-of-the-art deep learning techniques applied to this problem. By reviewing various approaches and evaluating their effectiveness, this paper contributes to the broader application of AI in precision agriculture. It guides future research and practical implementations that can help farmers reduce crop loss and manage fields more efficiently through advanced sensor technology.

In contribution 12, this survey paper explores the critical role of explainable AI (XAI) within the medical field, emphasizing the integration of AI with medical sensor data to improve diagnostics and patient care. By discussing recent progress and proposing new frameworks for XAI, the paper contributes to the ongoing development of transparent, understandable AI applications that can work alongside healthcare professionals. This work is especially important for enhancing the trustworthiness and efficacy of AI systems in interpreting complex medical sensor data, thus supporting better clinical decisions and patient outcomes.

## 3. Conclusions

The intersection of artificial intelligence (AI) and deep learning with sensor technologies presents an evolving frontier with profound implications across a myriad of applications, from healthcare diagnostics to autonomous systems and beyond. This collection of twelve papers contributes significant insights and innovations at this intersection, showcasing the potential of AI and deep learning in extracting, processing, and analyzing sensor data to solve complex real-world problems.

We appreciate all contributors of this Special Issue, as well as the reviewers of the submitted papers. Their dedicated efforts and expertise in providing thorough reviews greatly helped with the completion of this successful publication.
